# Supplementary material for: Exosomes released from senescent cells and circulatory exosomes isolated from human plasma reveal aging-associated proteomic and lipid signatures
Source: Aging (Albany NY). 2025 Jul 30;17(8):1929–65. doi: 10.18632/aging.206292 (PMC12422824; doi:10.18632/aging.206292)
Supplement: Supplementary Figures [file aging-17-8-206292-s001.pdf]

SUPPLEMENTARY FIGURES

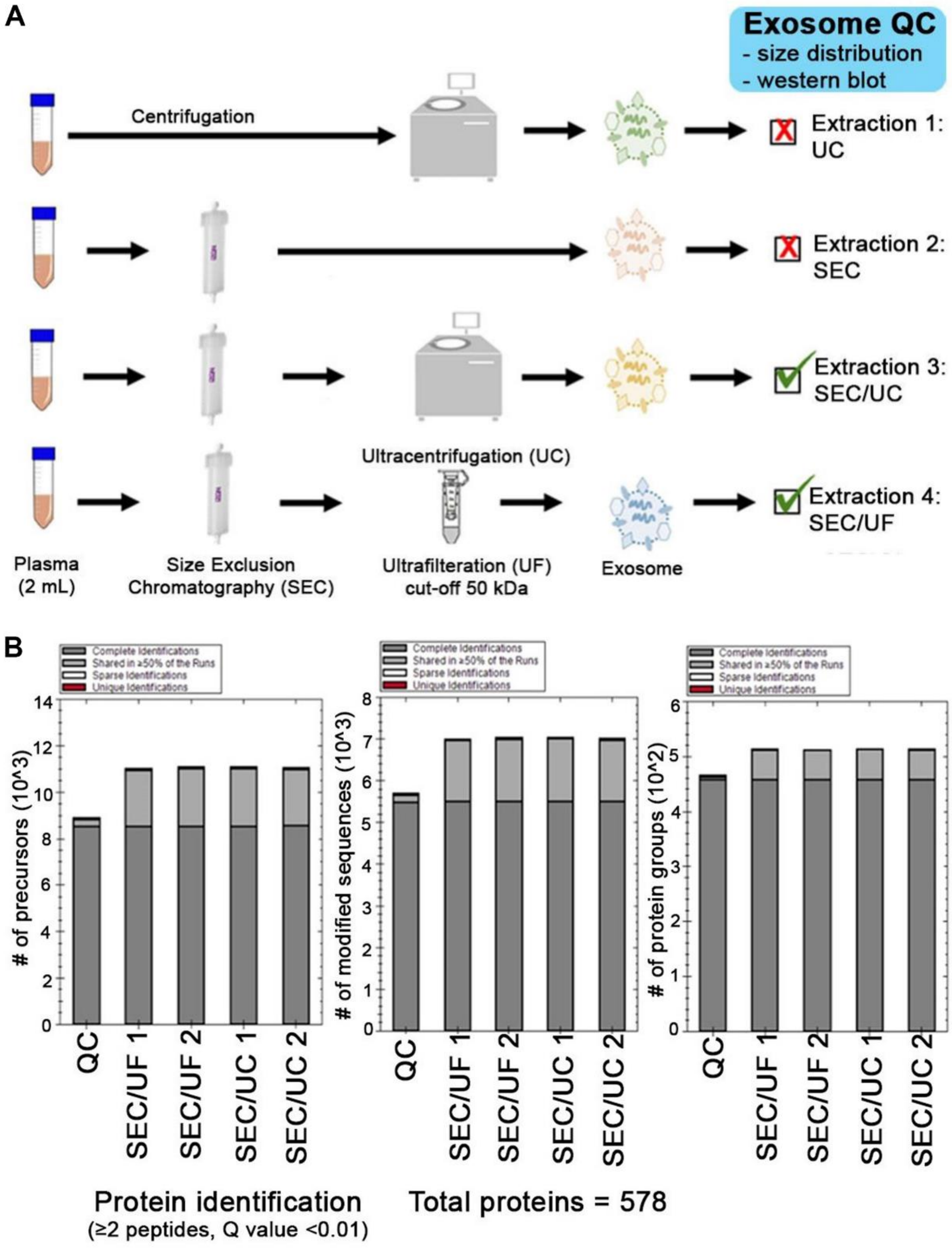

C

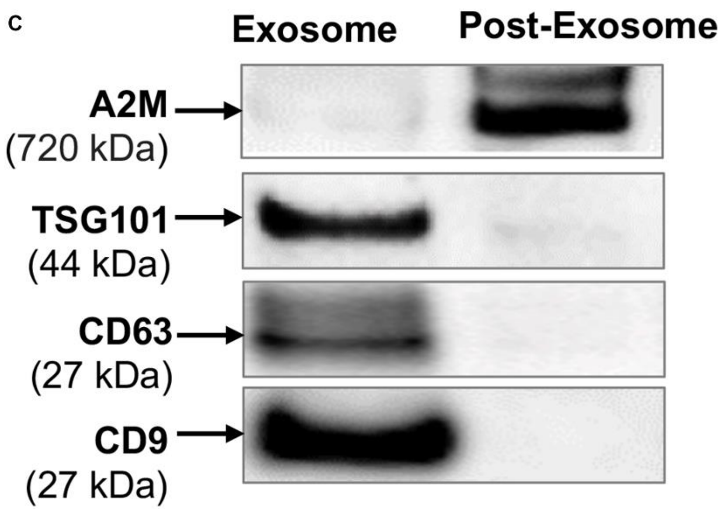

Supplementary Figure 1. (**A**) Workflow for the different plasma exosome enrichment methods were assessed for plasma exosome extraction. SEC: size exclusion chromatography, UC: ultracentrifugation, UF: ultrafiltration. (**B**) Bar graph showing the distribution of plasma exosome proteins identified using directDIA analysis in different exosome extraction methods UC and UF. UC: ultracentrifugation, UF: ultrafiltration. (**C**) Western blot confirms the presence of CD9, CD63, and TSG101 proteins (EVs markers) in cell culture extracted exosome, while the presence of Alpha-2-Macroglobulin (A2M) determined culture media protein contaminants.

A Spectral library details

Sample Details	Database	Precursors	Peptides	Proteins	Protein groups
25 DDA	HPRP fractionation dataset (DDA)	43,201	26,655	5,186	2,323
10 directDIA	directDIA dataset (DIA)	21,944	12,357	1,787	856
25 DDA + 10 directDIA	Hybrid dataset (DDA + DIA)	44,981	26,172	4,993	2,225

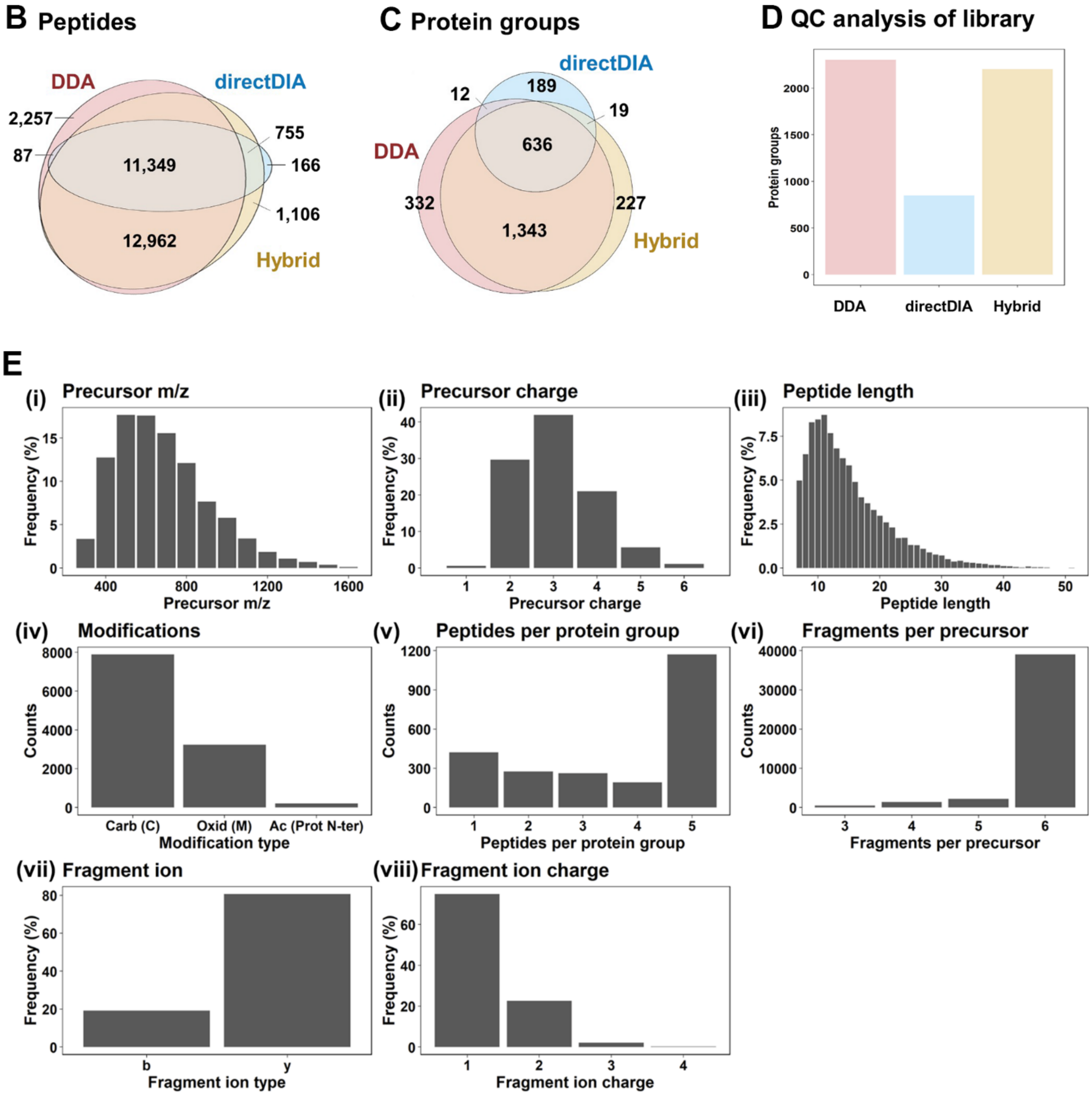

F

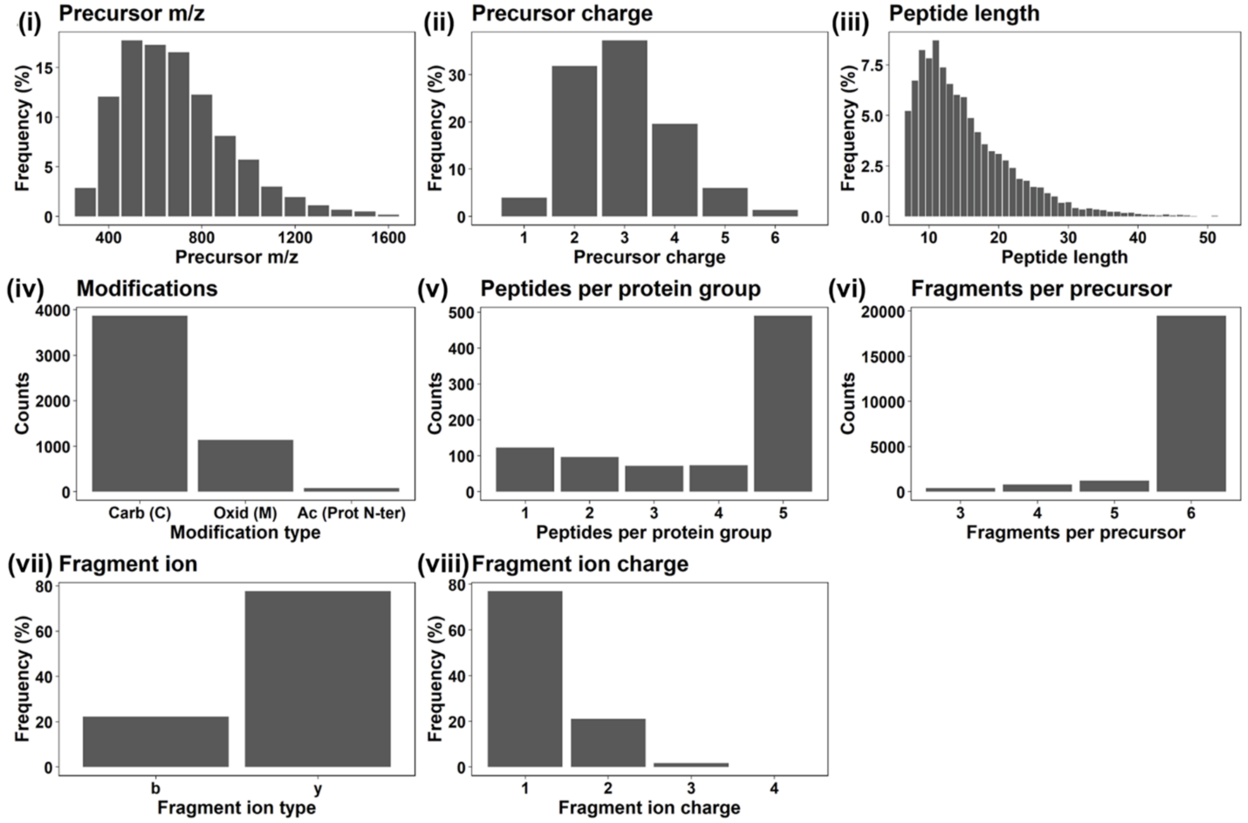
(i) Precursor m/z
Frequency (%)
Precursor m/z
(ii) Precursor charge
Frequency (%)
Precursor charge
(iii) Peptide length
Frequency (%)
Peptide length
(iv) Modifications
Counts
Carb (C)
Oxid (M)
Ac (Prot N-ter)
Modification type
(v) Peptides per protein group
Counts
Peptides per protein group
(vi) Fragments per precursor
Counts
Fragments per precursor
(vii) Fragment ion
Frequency (%)
b
y
Fragment ion type
(viii) Fragment ion charge
Frequency (%)
Fragment ion charge

G

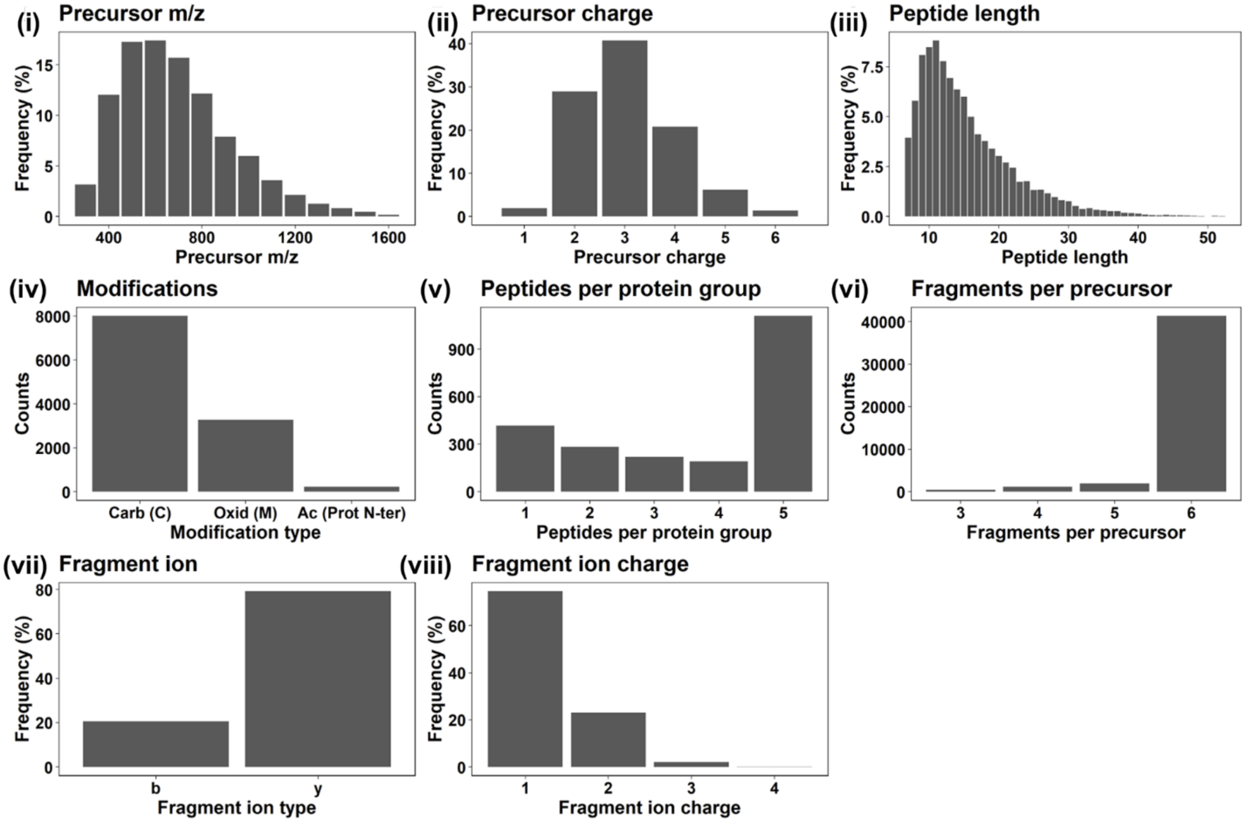
(i) Precursor m/z
Frequency (%)
Precursor m/z
(ii) Precursor charge
Frequency (%)
Precursor charge
(iii) Peptide length
Frequency (%)
Peptide length
(iv) Modifications
Counts
Carb (C)
Oxid (M)
Ac (Prot N-ter)
Modification type
(v) Peptides per protein group
Counts
Peptides per protein group
(vi) Fragments per precursor
Counts
Fragments per precursor
(vii) Fragment ion
Frequency (%)
b
y
Fragment ion type
(viii) Fragment ion charge
Frequency (%)
Fragment ion charge

H

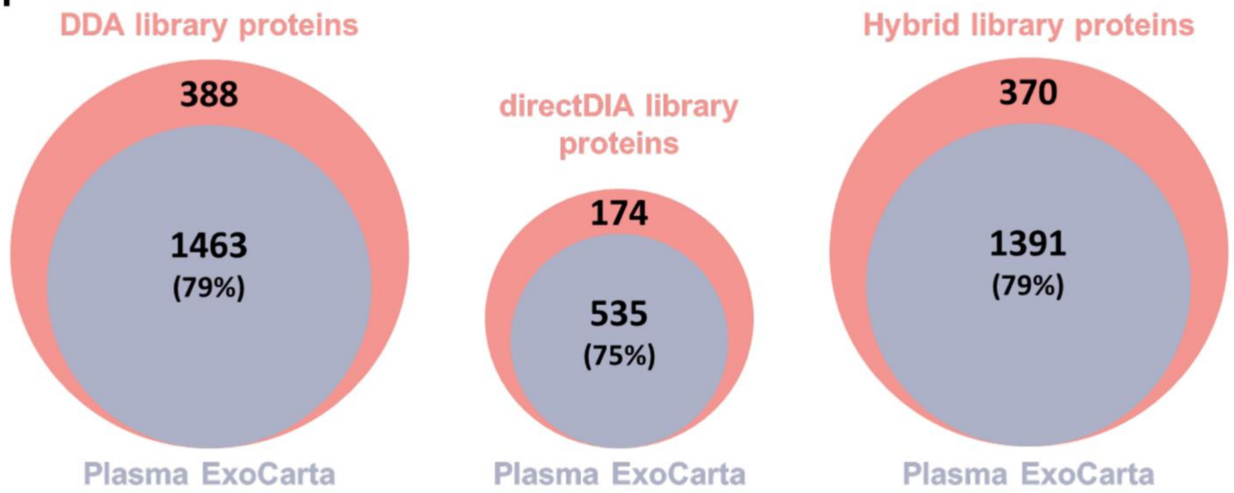

I

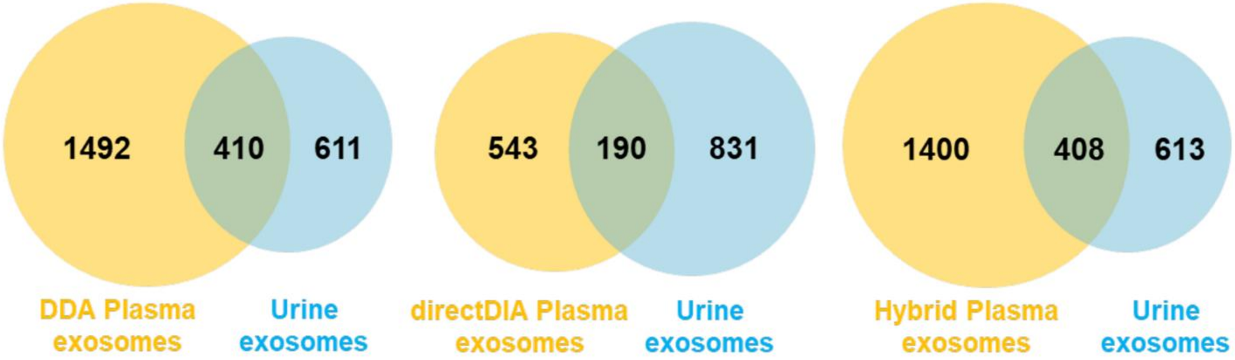

Supplementary Figure 2. (**A**–**D**) HPRP-fractionation and analysis of plasma exosomes for the generation of DDA, directDIA and hybrid-MS deep spectral proteomics. (**E**) Assessment of the DDA library. (i) Distribution of precursor m/z value. (ii) Distribution of precursor charge. (iii) Distribution of peptide length. (iv) Modifications of peptides. Carb (**C**), carbamidomethylation of cysteine residue; Oxi (M), oxidation of methionine residue; Ac (Prot N-ter), acetylation of protein N-terminus. (v) Number of peptides per protein group. (vi) Number of fragments per precursor. (vii) Fragment ion type. (viii) Fragment ion charge. (vi, vii, viii) Only fragments used in the assay were considered. (**F**) Assessment of the directDIA library. (i) Distribution of precursor m/z value. (ii) Distribution of precursor charge. (iii) Distribution of peptide length. (iv) Modifications of peptides. Carb (C), carbamidomethylation of cysteine residue; Oxi (M), oxidation of methionine residue; Ac (Prot N-ter), acetylation of protein N-terminus. (v) Number of peptides per protein group. (vi) Number of fragments per precursor. (vii) Fragment ion type. (viii) Fragment ion charge. (vi, vii, viii) Only fragments used in the assay were considered. (**G**) Assessment of the hybrid library. (i) Distribution of precursor m/z value. (ii) Distribution of precursor charge. (iii) Distribution of peptide length. (iv) Modifications of peptides. Carb (**C**), carbamidomethylation of cysteine residue; Oxi (M), oxidation of methionine residue; Ac (Prot N-ter), acetylation of protein N-terminus. (v) Number of peptides per protein group. (vi) Number of fragments per precursor. (vii) Fragment ion type. (viii) Fragment ion charge. (vi, vii, viii) Only fragments used in the assay were considered. (**H**) Venn diagrams showing the common and unique protein groups in the plasma exosome libraries (DDA, directDIA and hybrid; $\geq$ 2 unique peptides) and the human ExoCarta database. Gene names were used to generate the Venn diagrams. (**I**) Venn diagrams showing the common and unique exosome protein groups in the plasma exosomes libraries (DDA, directDIA and hybrid; $\geq$ 2 unique peptides) and the urine exosome library. UniProt IDs were used to generate the Venn diagrams.

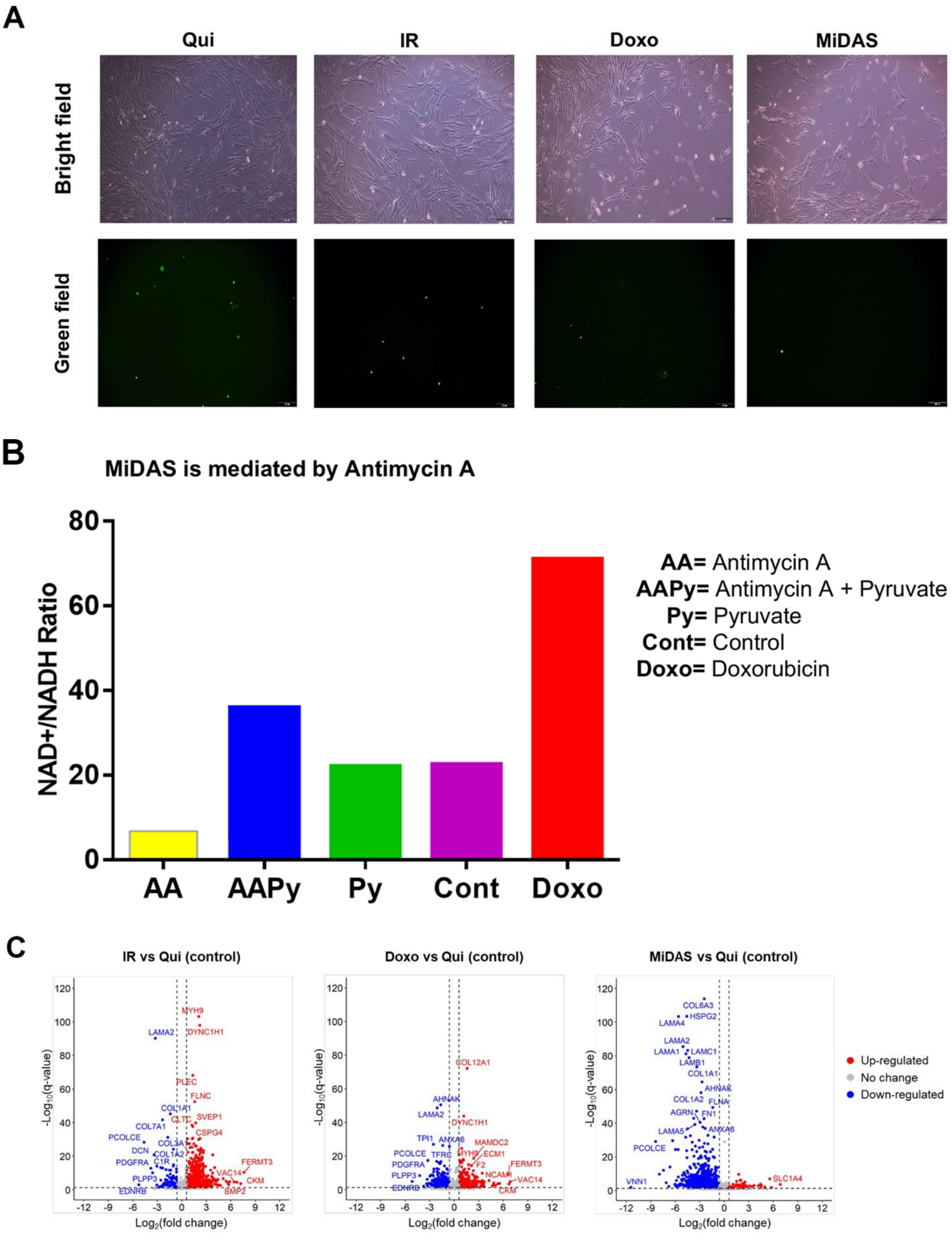

Supplementary Figure 3. (**A**) Estimation of cell death using SYTOX Green Cytotoxicity Assay. (**B**) NAD+/NADH ratio calculations for IMR90 cells. (**C**) Volcano plots showing the significantly altered protein groups in senescence (IR, Doxo, MiDAS) vs Quiescent (control) out of the 1,426 quantifiable protein groups (q-value ≤ 0.05, |log2fold change| ≥ 0.58).

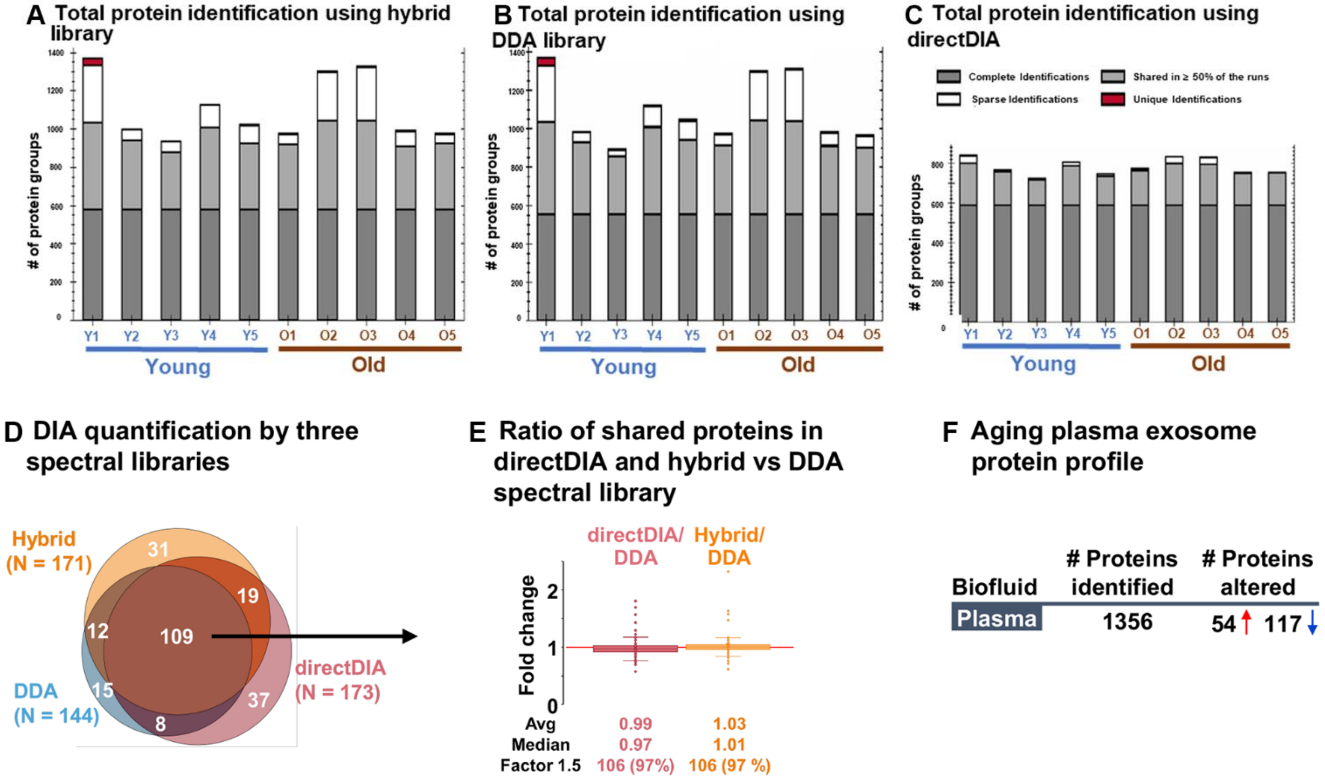

Supplementary Figure 4. (**A**–**C**) Bar diagrams showing the total number of identified and quantified protein groups in the human plasma exosome study with the three different libraries (hybrid, DDA, and directDIA). (**D**) Venn diagram showing the common and unique significantly altered human plasma exosome protein groups obtained with the three different libraries. (**E**) Boxplot showing the ratio of the 'Old vs Young' fold-change for the shared significantly altered protein groups obtained with directDIA or hybrid library vs DDA library. (**F**) Summary of all quantifiable (with $\geq$ 2 unique peptides) and significantly altered age-specific plasma exosome protein groups (q-value < 0.05, |log2 fold change| $\geq$ 0.58). We used DIA-MS protein identification and quantification to analyze plasma exosomes from young and elderly individuals and processed the data using three different spectral libraries: hybrid DDA-DIA library, DDA library, or directDIA library. Overall, we reproducibly identified and quantified a total of 1,356 protein groups using the hybrid spectral library (Supplementary Figure 4A and Supplementary Table 4B), 1,349 protein groups using the spectral library generated from DDA searches (Supplementary Figure 4B and Supplementary Table 4E), and 760 protein groups when applying directDIA searches (Supplementary Figure 4C and Supplementary Table 4H). For all, we reported protein groups numbers obtained with a 1% false discovery rate (FDR) and ≥ 2 unique peptides. We investigated the significantly changing protein groups in plasma exosomes from 'old vs young' individuals that resulted from all spectral library-based approaches, and interestingly 109 significantly altered proteins were commonly shared between all libraries (q-value < 0.05 and |log2 old vs young| ≥ 0.58; Supplementary Figure 4D). The highly consistent fold-changes of the shared protein groups with the hybrid and directDIA library compared to the DDA spectral library demonstrate that all spectral library workflows are robust and can be used for protein quantification (Supplementary Figure 4E). However, the maximum depth of coverage was obtained with the hybrid spectral library with 1,356 identified and quantified protein groups (≥ 2 unique peptides), so we focused on the 171 protein groups that significantly changed within plasma exosomes between the 'old vs young' cohorts, when using the DIA-MS hybrid library approach (Supplementary Figure 4F), in this study.

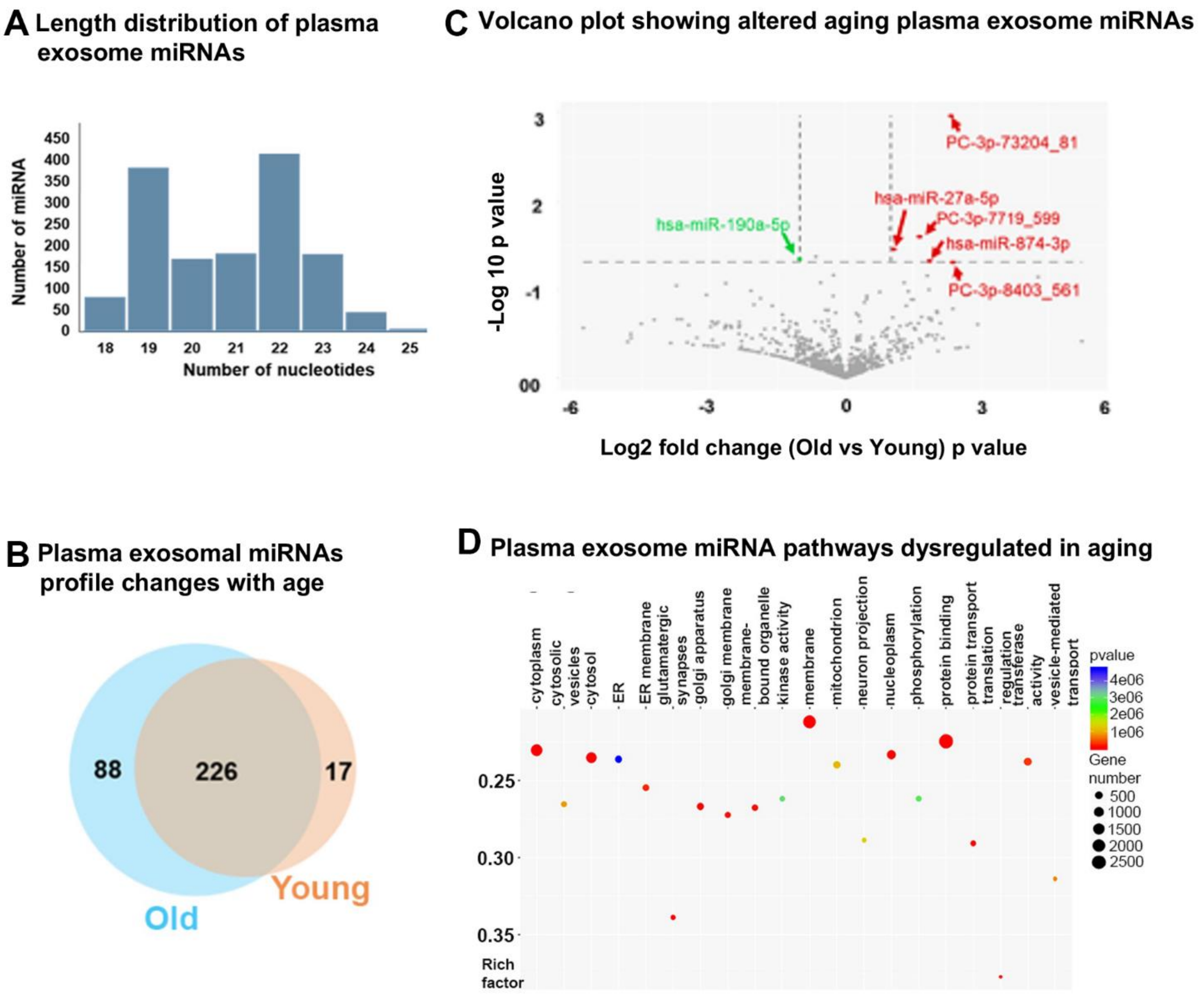

Supplementary Figure 5. Age-specific plasma exosome miRNA signatures in old and young cohorts. (**A**) miRNA nucleotide size distribution. (**B**) Overlapping and unique exosome miRNAs in plasma from older and young individuals. (**C**) Volcano plot showing significantly altered age-specific plasma exosome miRNAs (P-value < 0.05 and > 1.5-fold). Red, upregulated; Green, down-regulated; and Gray, not significantly changed. A few differentially abundant miRNAs are labeled. (**D**) Age-related exosome miRNA pathways.

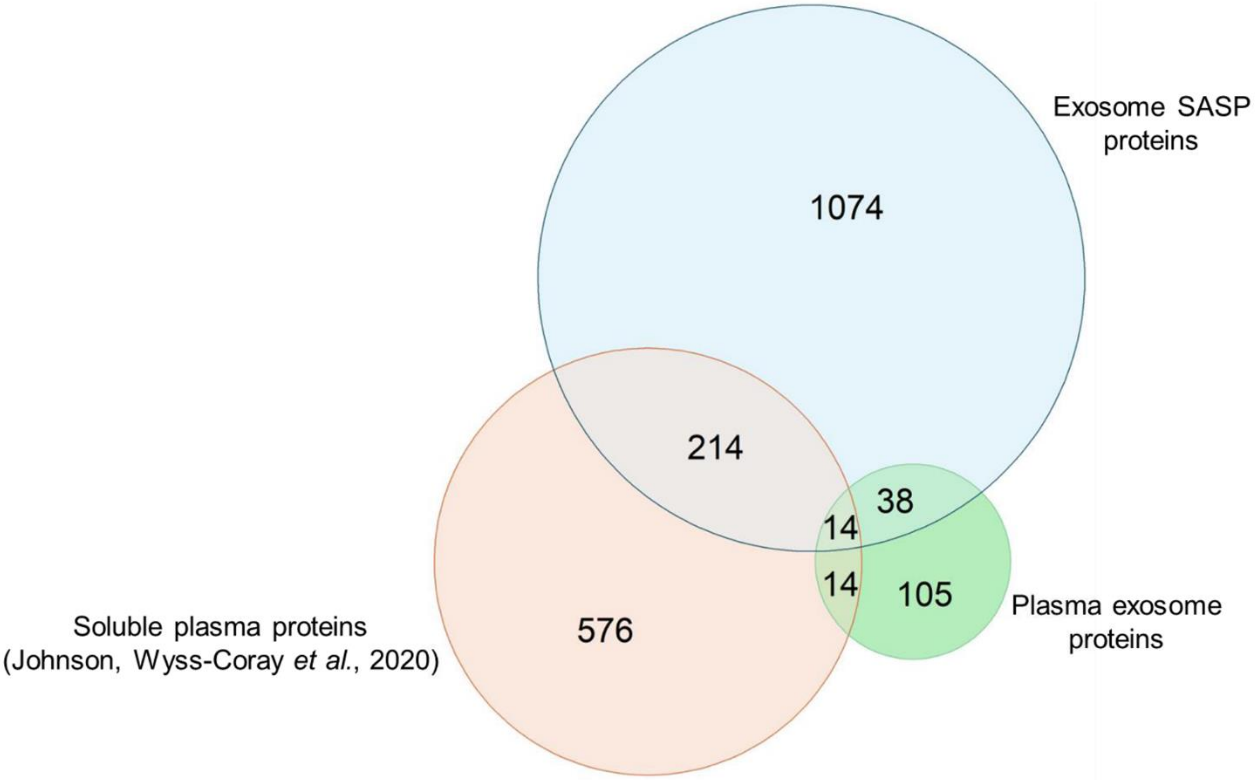

Supplementary Figure 6. Venn diagram showing the common and unique significantly altered protein groups in human plasma exosomes from this study (old vs young comparison, "Plasma exosome proteins"), in senescence-induced IMR90 exosomes (merged from the three inducers) from this study ("Exosome SASP proteins"), and human age-associated soluble plasma proteins from Johnson, Wyss-Coray et al., 2020 ("Soluble plasma proteins") [32].
