# Supplementary material for: Exosomes released from senescent cells and circulatory exosomes isolated from human plasma reveal aging-associated proteomic and lipid signatures
Source: Aging (Albany NY). 2025 Jul 30;17(8):1929–65. doi: 10.18632/aging.206292 (PMC12422824; doi:10.18632/aging.206292)
Supplement: Supplementary Table 1 [file aging-17-8-206292-s002.pdf]

SUPPLEMENTARY TABLE

Supplementary Table 1. Plasma sample details.

Sample	Sample ID	Age (yrs)
Young 1	Y1	24
Young 2	Y2	26
Young 3	Y3	26
Young 4	Y4	20
Young 5	Y5	22
Old 1	O1	74
Old 2	O2	67
Old 3	O3	70
Old 4	O4	65
Old 5	O5	65
